# Identification and Characterization of the RNA Modifying Factors PUS7 and WTAP as Key Components for the Control of Tumor Biological Processes in Renal Cell Carcinomas

**DOI:** 10.3390/cimb47040266

**Published:** 2025-04-09

**Authors:** Tim Hohmann, Urszula Hohmann, Faramarz Dehghani, Olaf Grisk, Simon Jasinski-Bergner

**Affiliations:** 1 Department of Anatomy and Cell Biology, Medical Faculty, Martin Luther University Halle-Wittenberg, Grosse Steinstrasse 52, 06108 Halle (Saale), Germany; tim.hohmann@uk-halle.de (T.H.); urszula.hohmann@uk-halle.de (U.H.); faramarz.dehghani@medizin.uni-halle.de (F.D.); 2 Institute of Physiology, Brandenburg Medical School (MHB), Theodor Fontane, Hochstraße 29 Haus 11 2.OG, 14770 Brandenburg an der Havel, Germany; olaf.grisk@mhb-fontane.de

**Keywords:** RCC, transcriptome, PUS7, WTAP, alternative splicing, RNA modifications

## Abstract

Current research discusses the putative importance of RNA modification in tumor diseases. These RNA modifications include predominantly pseudouridinylation, ortho-methylations on the ribose residues, as well as methylations on the organic bases. Such chemical modifications directly influence fundamental properties such as transcript stability, alternative splicing, and translation efficiency, all of which are basic requirements for (tumor) cell proliferation, cell metabolism, cell migration, apoptosis resistance, etc. In this comparative study, the two RNA-modifying factors, pseudouridine synthase 7 (PUS7, RNA pseudouridinylation) and WT1-associated protein (WTAP, m6A RNA methylation), were identified using data from human renal cell carcinoma (RCC) tumors. PUS7 and WTAP showed a statistically significant correlation with relevant proliferation and prognosis markers such as CXCR4, TP53, PTEN, and NRAS, as well as with the two tumor immune checkpoints HLA-G and LGALS9 and were directly associated with a statistically significant effect on overall survival. Furthermore, comparative analyses also identified further putative target mRNAs of importance for tumor biology of PUS7 and WTAP. In particular, components with direct relevance for mitosis, the cell cycle, and cell division, as well as the WNT pathway, were identified.

## 1. Introduction

The heterogeneous and complex group of tumor diseases was initially simply described as a growing mass of tissue, as a so-called tumor [1]. Current and ongoing research has resulted in this term being fundamentally refined. In particular, the defined Hallmarks of Cancer by Douglas Hanahan and Robert A. Weinberg [2] meanwhile clearly summarize the complexity of these diseases and thereby reflect the high level of scientific knowledge. These hallmarks include the autonomous maintenance of proliferation, including replicative immortality, evasion of growth suppressors, resistance to apoptosis, the ability to autonomously induce angiogenesis, immune evasion, genomic instability, and the accumulation of mutations and epigenetic reprogramming, deregulated cellular metabolism with effects on the patient’s microbiome, increased migration, and tissue invasion, but also phenotypic plasticity of tumor cells [2,3,4]. However, these multiple molecular causes may act independently from each other, also taking into account a possible relevant temporal order, or probably these mechanisms induce themselves, building on each other. With the progressive accumulation of these molecular dysregulations, cumulative additive effects also arise, increasing malignancy. Anyhow, they can mostly be broken down to defects and dysregulations on DNA and RNA levels.

In addition to transcription, translation is also a limiting factor in proliferation. In-deed, it has already been demonstrated that the translation efficiency (TE) of a cell is directly linked to proliferation, metabolism, and viability of cells and per se based upon the number of cellular ribosomes [5,6]. The more ribosomes are located on a coding transcript, the more efficient the process of translation is. Therefore, there exists a direct proportionality of TE to the ribosomal density in the cells [7,8]. An increase in ribosomes after malignant transformation has been observed in various tumor diseases. Furthermore, the size of nucleoli, which represent the major location of ribosomal assembly, has been an important prognostic parameter in pathology to distinguish between benign and malignant tumors as well as being of prognostic value [9]. During these molecular biological processes of translation, the different RNA species interact in a regulatory manner within a network.

The human transcriptome includes the entirety of all RNA molecules transcribed from DNA within a human cell. Within an organism, there are corresponding differences in the transcriptome between cells of various tissues, cell types within a tissue, developmental states (e.g., age, differentiation), and distinct physiological and pathophysiological states (including exemplarily malignant transformation) [10]. The three main types of RNA involved in protein biosynthesis include messenger RNA (mRNA), transfer RNA (tRNA), and ribosomal RNA (rRNA) [11].

In healthy cells, the fractions of the three RNA species relevant for protein biosynthesis are approximately as follows: 80–90% rRNA of total RNA, 10–15% tRNA, and 1–5% mRNA. These percentages refer to the proportion of the total RNA mass. If one were to consider the number of molecules, tRNA molecules would represent the absolute majority [12]. The process of malignant transformation can lead to a strong shift in this composition mediated by altered RNA modifications [13]. However, there are also other RNA species that perform more regulatory functions, for example, those involved in post-transcriptional modifications or gene regulation as well as in DNA replication. These include the small nuclear RNAs (snRNAs), microRNAs (miRNAs), YRNAs, and long non-coding RNAs (lncRNAs) [12].

Interestingly, rRNAs, tRNAs, and mRNAs undergo several chemical modifications for their stabilization, which also affects their secondary structures with further consequences for their full functionality per se. At this point, RNA pseudouridinylation and RNA methylation should be mentioned in particular, which represent common RNA modifications of these RNA species with a corresponding influence on these properties.

To further investigate the significance of the most common chemical RNA modifications on tumor biology, especially with regard to translation efficiency and thus indirectly on proliferation as well as on malignancy, the three most frequent subtypes of renal cell carcinoma (RCC) should be analyzed as a model system. Renal cell carcinomas (RCCs) are the most common type of cancer arising in the kidney with certain risk factors, including, among others, smoking, obesity, high blood pressure, and exposure to cancerogenic chemicals such as trichloroethylene [14]. About 90% of these renal tumors arise from the renal tubular epithelial cells, with the respective most common subtypes: clear cell RCC (ccRCC), papillary RCC (pRCC), and chromophobe RCC (chRCC). These different subtypes differ distinctly in various parameters, such as proliferation, metastasis, survival rates, and treatment options [15]. With an incidence of 75% of RCCs, ccRCC is the largest subtype, which arises from epithelial cells of the proximal tubule of the nephron and is characterized by particularly high aggressiveness and high proliferation, while pRCC (incidence of 10%, linked to distal tubular epithelium) and chRCC (incidence of 5%, to the intercalated cells of the collecting duct). Although pRCC is also characterized by high aggressiveness, the proliferation of these tumor cells tends to be lower than that of ccRCCs, whereas chRCCs have a comparatively lower mortality and proliferation than the other two subtypes [16].

In contrast to the histological subdivisions of these subtypes, the genetic differences between these three RCC subtypes are more difficult to determine. However, it should be emphasized that in 95% of ccRCCs, the loss of the short arm of chromosome 3 (3p), where the *VHL* gene is located (3p25.3), can be observed, which affects the transcription factor HIF-1alpha and its thereby induced gene expression. These even include growth factors such as VEGF and PDGF [17].

Current research is concerned with the relevance of RNA modifications in tumor diseases. However, corresponding studies are still lacking. In this study, the expression data of the most important RNA-modifying proteins in RCC TCGA data sets were examined to elucidate to which extent their gene expression patterns correlate with the known pathological parameters of proliferation and malignancy of the three RCC tumor entities. In addition to known marker genes for proliferation and prognosis in RCCs, tumor immune checkpoint molecules relevant to RCC therapy were also analyzed for putative correlations to factors involved in major RNA modifications.

## 2. Materials and Methods

### 2.1. Data Sets

For the analysis, the TCGA-KIRC, TCGA-KICH, and TCGA-KIRP RNAseq data sets were used [18,19,20]. These consist of 1028 cases in total, of which 91 were chromophobe RCC, 323 papillary RCC, 600 clear cell RCC, and 14 cases of RCC belonging to none of those tumor types. Furthermore, 18 patients received previous treatment, potentially altering the expression profile. Thus, those 18 cases and the 14 cases belonging to none of the three main classifications were excluded from further analysis, leaving 91 chromophobe RCC, 323 papillary RCC, and 582 clear cell RCC for analysis.

Additionally, non-tumorous kidney samples from the CPTAC-3 data set were used for analyzing correlation coefficients between genes of interest. This set contained 102 samples.

For the validation of correlations, the GSE15641 [21], GSE17818 [22,23,24], and GSE17895 [22,23,24] microarray datasets of kidney tumors were used. These sets were reduced to all kidney tumors, being either classified as clear cell, papillary, or chromophobe RCC, corresponding to 49, 102, or 138 RCC.

### 2.2. Data Analysis

The analysis of differential expression was performed using Matlab R2021a with the DESeq2 approach (https://github.com/jbmorlot/DESeq2-matlab?tab=readme-ov-file, accessed on 12 June 2023) [25], as described previously [26,27]. Genes were assumed to be differentially expressed, if the false discovery rate was smaller than 0.05. Differential expression was analyzed between all three tumor subgroups individually. Alternatively, the GENT2 database (http://gent2.appex.kr/gent2/, accessed on 12 June 2023) [28] was used to assess the relative expression of PUS7 and WTAP between multiple studies of kidney cancer and healthy kidneys. To assess the correlation between the different genes of interest (see Table 1), the transcripts per million (TPM) values of genes of interest were correlated using the Spearman rank correlation coefficient. To estimate the robustness of the correlation coefficient, bootstrapping was employed, resampling the data set 10,000 times to obtain empirical 95% confidence intervals. For analysis and visualization of patient survival, the MatSurv tool was used [29]. The grouping of cases in high and low expressing patients was based on a median split, and the log-rank (Mantel-Cox) test was used to compare the survival distributions.

For analysis of correlations of relevant genes identified in the previous steps with the remaining transcriptome, all samples from each RCC subtype were split according to their median into a high and low expressing group, and differential expression between high and low expressing RCC of one group was analyzed via DESeq2, as above. The obtained gene list was restricted afterwards to those genes that were differentially expressed in all three subtypes. The results were then plotted as a heatmap, sorted by the expression given as z-scores of the gene of interest along the *x*-axis and the respective correlation coefficient for each differentially expressed gene (*y*-axis) that showed correlation with the gene of interest with correlation coefficients ≥0.3 or ≤−0.3. Functional enrichment of differentially expressed genes was assessed using https://davidbioinformatics.nih.gov/ (accessed on 12 June 2023) [30,31].

## 3. Results

### 3.1. Tabular Overview of Analyzed Genes

In this project, known RNA-modifying enzymes involved in RNA pseudouridinylation, RNA ortho-methylation, and RNA N-methylation, as well as selected genes with strong relevance for tumor (immuno) biology, were analyzed in RCC tumors. Table 1 summarizes the investigated genes and Supplementary Table S2 lists the full gene names.

### 3.2. Analysis of RCC-Relevant Proliferation and Prognostic Marker Genes

In molecular diagnostics, the expression of certain marker genes with relevance for proliferation and prognosis is often used to assess the aggressiveness of tumors and to estimate suitable treatment options. The following proliferation and prognostic markers are frequently used in RCC diagnostics: MKI67, PCNA, MCM2, MCM4, CENPF, CXCR4, and TP53, BCL2, BIRC5, PTEN, NRAS, TSC1, TSC2, and CDKN2A [32,33,34,35,36]. Considering that the following order exists with regard to the proliferation and malignancy of the 3 RCC subtypes: ccRCC > pRCC > chRCC, the proliferation and prognosis markers typical for RCC were analyzed in the respective TCGA data sets (ccRCC *n* = 582; pRCC *n* = 323, chRCC *n* = 91). The detection of differentially expressed genes was carried out by comparing the three different RCC subtypes with each other. First, the particularly fast-proliferating and aggressive ccRCC was compared with the chRCC and subsequently with the pRCC, and then afterwards the comparison between pRCC and chRCC was performed.

**Figure 1:**
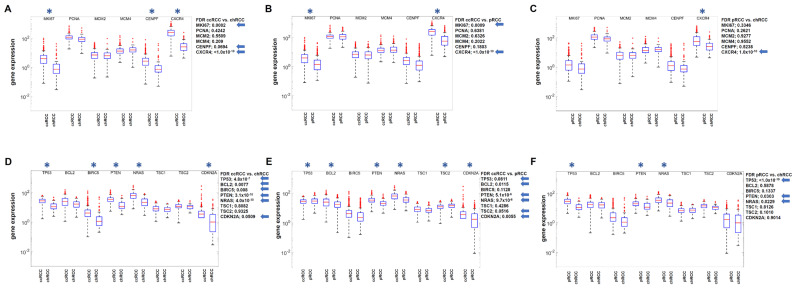
Comparative analysis of DESeq2 normalized gene expression for cell proliferation markers (**A**–**C**) and prognostic markers (**D**–**F**) between the three RCC subtypes according to the order ccRCC > pRCC > chRCC with regard to proliferation and malignancy. Statistically significant differences in gene expression (FDR) are marked with an asterisk and the exact numerical values on the side with an arrow. Blue color indicates downregulation and red color upregulation.

We observed that the proliferation marker CXCR4, in particular, showed a statistically significant difference in gene expression according to this ranking of the RCC subtypes (Figure 1A–C). Furthermore, the prognostic markers TP53, PTEN, and NRAS were also identified as statistically significant differentially expressed according to the ranking of the RCC subtypes (Figure 1D–F).

### 3.3. Validation of Selected Immune Checkpoint Axes in the RCC Data Sets

**Figure 2:**
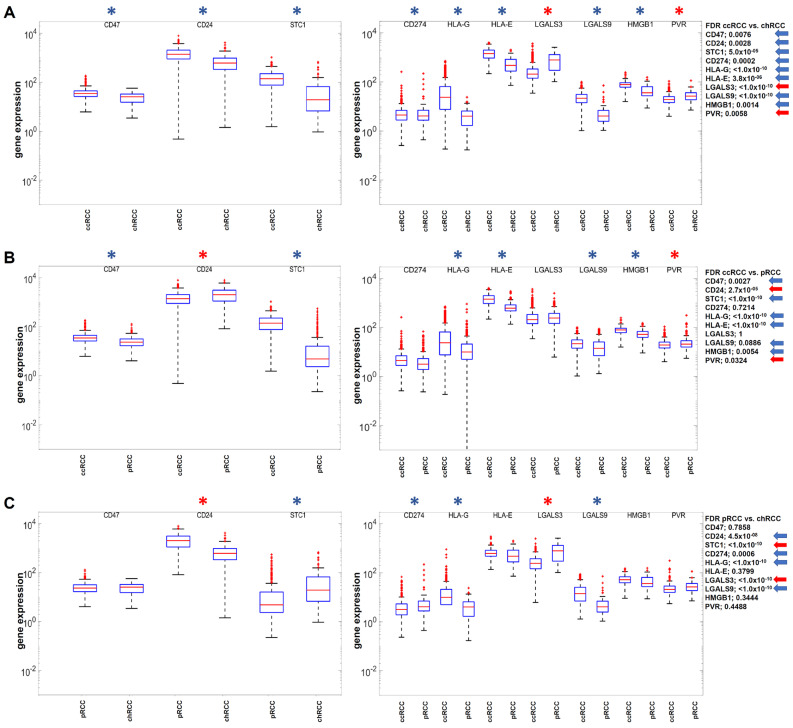
Comparative analysis of DESeq2 normalized gene expression for immune checkpoint axes (**A**–**C**) with available therapeutic mAbs between the three RCC subtypes according to the order ccRCC > pRCC > chRCC regarding proliferation and malignancy. Statistically significant differences in gene expression (FDR) are marked with an asterisk, and the exact numerical values are marked with an arrow on the side. Blue color indicates downregulation and red color upregulation.

Antibody-based tumor immune checkpoint axis blockades have gained immense importance for RCC therapies with promising overall response rates. Therefore, the relevant checkpoint molecules, for which therapeutic mAbs are available, were analyzed in the RCC settings as well. Frequently, such immune checkpoint molecules contribute to tumor immune evasion and thus indirectly to proliferation and malignancy within the patient. It was shown that in particular the gene expression of HLA-G and LGALS9 matched statistically significantly with the in vivo characterizations of the three RCC subtypes (Figure 2A–C).

### 3.4. Identification of Differentially Expressed Genes Relevant for RNA Pseudouridinylation and RNA Methylation in the RCC Data Sets

**Figure 3:**
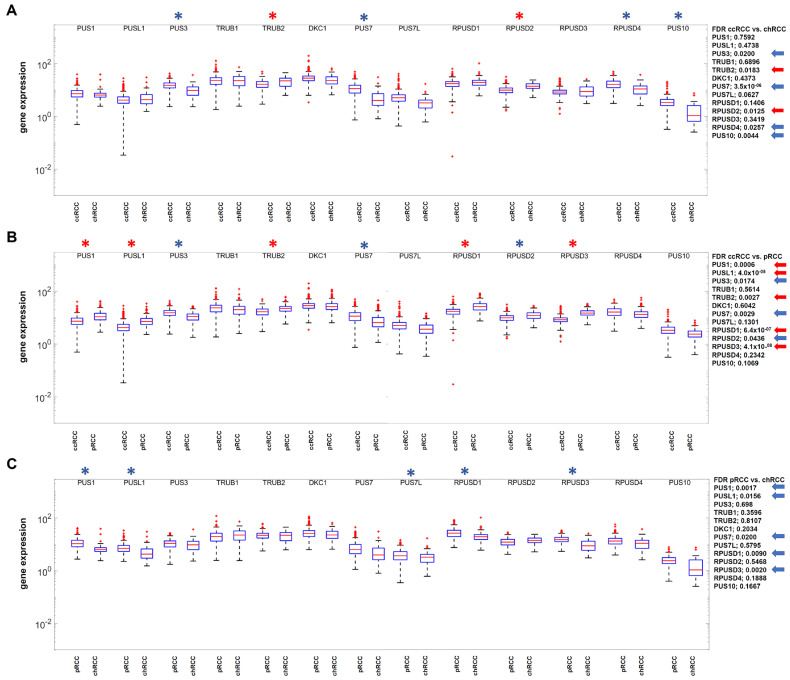
Comparative analysis of DESeq2 normalized gene expression for factors with relevance for RNA pseudouridinylation (**A**–**C**) in the three RCC subtypes according to the order ccRCC > pRCC > chRCC with respect to proliferation and malignancy. Statistically significant differences in gene expression (FDR) were marked with a star, and the exact numerical values were marked with an arrow on the side. The blue color was used for downregulation and the red color for upregulation.

**Figure 4:**
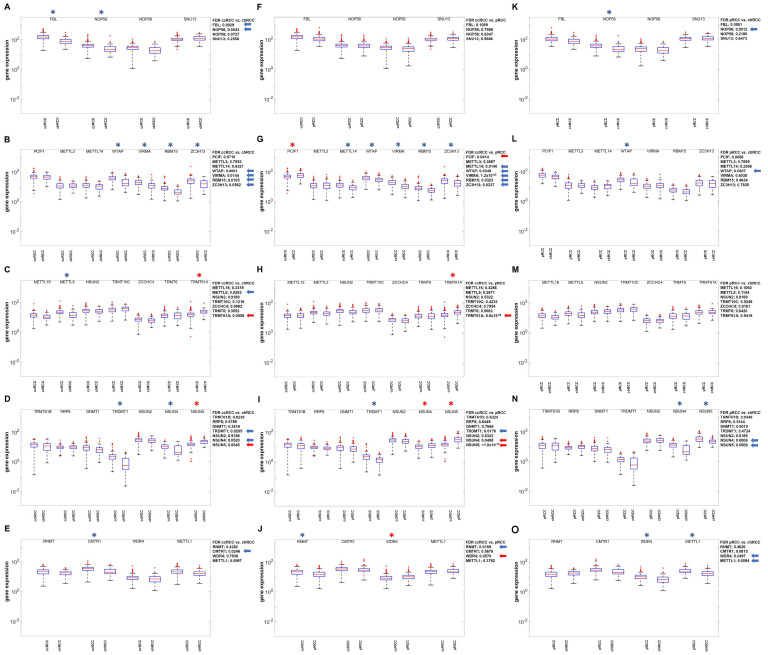
Comparative analysis of DESeq2 normalized gene expression for factors relevant to RNA ortho-methylations (**A**,**F**,**K**) and methylations on the organic bases (**B**–**E**,**G**–**J**,**L**–**O**) in the three RCC subtypes according to the order ccRCC > pRCC > chRCC with regard to proliferation and malignancy. Statistically significant differences in gene expression (FDR) were marked with a star, and the exact numerical values were indicated with an arrow on the side. The blue color was used for down-regulation and the red color for up-regulation.

To analyze the hypothesis of the significance of RNA-modifying enzymes for proliferation and prognosis as putative limiting bottlenecks in tumor diseases, the next step was to determine the differential gene expression of all known RNA-modifying proteins for pseudouridinylation and methylation (Figure 3 and Figure 4).

While in the case of RNA pseudouridinylation, consistently statistically significant changes could not be detected when comparing the gene expressions of the three RCC subtypes, two different factors involving RNA methylation were identified as statistically differentially expressed, namely PUS7 and WTAP (Figure 3).

### 3.5. Examination of Putative Correlations Between the Two RNA-Modifying Factors PUS7 and WTAP as Well as the Differentially Expressed Marker Genes Relevant for Tumor (Immune) Biology

**Figure 5:**
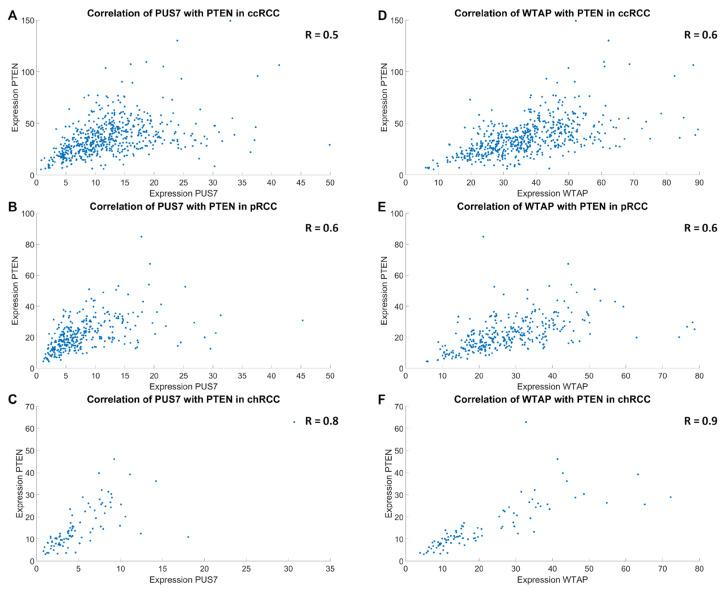
Exemplary visualization of the correlations of PTEN with PUS7 (**A**–**C**) and WTAP (**D**–**F**) in the three RCC subtypes. Expressions are given in transcripts per million reads.

The next step was to investigate the extent to which the marker genes previously found to be statistically significantly differently expressed in the RCC settings correlate with the RNA-modifying factors PUS7 and WTAP. It was shown that, in all RCC subtypes and in non-tumorous kidneys (but particularly strong in chRCCs), there is a statistically significant high to very high positive correlation with the prognostic markers TP53, PTEN, and NRAS (Table 2, Figure 5). In addition, a statistically significant, weak positive correlation was observed in the different RCC subtypes and kidney specimen with the proliferation marker CXCR4 as well as with the two tumor immune checkpoint molecules HLA-G and LGALS9 (Table 2). Of note, the average expression in kidney tumor specimens for PUS7 was 1.4× higher (*p* < 0.001) than in the non-tumorous kidneys or 1.2× higher for WTAP (*p* < 0.001), according to data from the GENT2 database [28]. To verify these results, the GSE15641 [21], GSE17818 [22,23,24], and GSE17895 [22,23,24] data sets were analyzed regarding the correlation of PUS7 and WTAP with the prognostic, proliferative, and immune checkpoint markers. There, we could verify most correlations, most notably with PTEN, CXCR4, and HLA-G in at least two of three data sets. For all others but TP53, we could find comparable correlations in at least one data set (Supplementary Table S1). Only for TP53 we could not find similar correlations at all. Nonetheless, this agreement verifies the analysis and most of the results as potentially biologically relevant.

**Figure 6:**
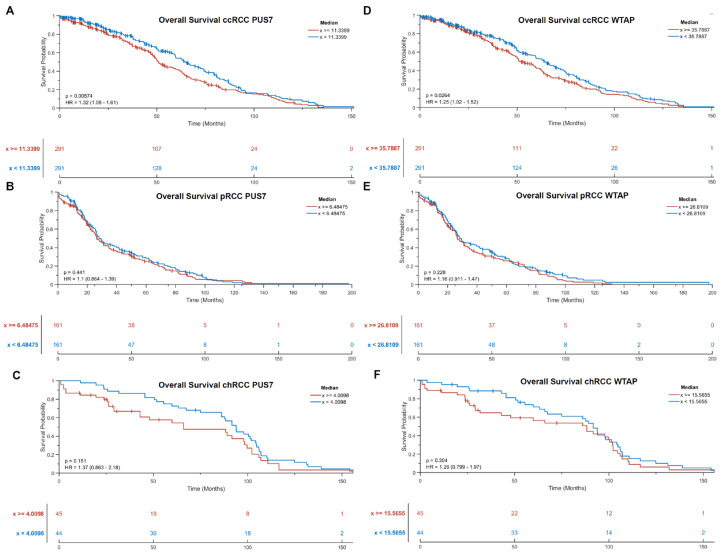
Investigation of the effect of PUS7 (**A**–**C**) and WTAP (**D**–**F**) on overall survival in the three RCC subtypes. The division into high versus low was done by median split of TPM expression values and analyzed with the log-rank (Mantel-Cox) test using the MatSurv tool [29].

Due to the fact that there is a statistically significant high to very high positive correlation with the three prognostic markers and the RNA-modifying factors PUS7 and WTAP, a putative influence of these two RNA-modifying factors on the overall survival in the three RCC subtypes was analyzed using Kaplan–Meier plots. It was shown that for the largest subgroup of ccRCCs, which is the most aggressive and the most common subtype of RCCs, there is a statistically significant difference in overall survival. Increased expression of PUS7 (*p* = 0.0057) and WTAP (*p* = 0.0264) is associated with poorer overall survival. This effect was also visible in the other two subtypes but was not statistically significant (Figure 6).

### 3.6. Identification of Additional PUS7 and WTAP Target Genes in RCCs

To clarify the observed statistically significant effect on survival in ccRCCs compared to the other two RCC subtypes, comparative transcriptome analyses were performed for PUS7 and WTAP with the aim of identifying the dysregulated target genes in the comparison of PUS7 high versus PUS7 low or WTAP high versus WTAP low tumors. High and low expressing samples were identified based on a median split, identical to the survival analysis.

**Figure 7:**
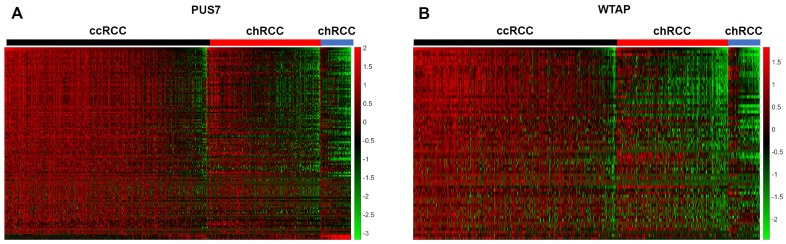
Heatmap visualizations show gene expression patterns of putative target genes that were statistically significantly altered when comparing PUS7 high versus low (**A**) and WTAP high versus low (**B**) in the three RCC subtypes. Furthermore, these target genes had to fulfill the following criteria: the correlation coefficients had to be either ≥ 0.3 or ≤−0.3 with PUS7 (**A**) and WTAP (**B**) in all three RCC subtypes.

The statistically significantly up- or down-regulated transcripts in the comparison of PUS7 high versus low expressers and, in analogy, for WTAP were shown as heatmaps for all three RCC subtypes. Between the three RCC subtypes, different expression levels of dysregulated transcripts can be seen (Figure 7). Notably, for almost all of the identified genes the correlation coefficient with PUS7 or WTAP was positive and only five genes in each group (≈10% for PUS7 and ≈5% for WTAP) were negatively correlated, suggesting the possibility of a large cluster of co-regulated genes. In all three RCC subtypes, there were a large number of YRNAs among the statistically significant dysregulated transcripts, which also correlated statistically significantly with the expression of PUS7 and WTAP. This is very interesting and important for the analysis per se, because YRNAs play an eminent role in DNA replication, are components of ribonucleoproteins, and are especially involved in RNA quality control [37]. The coding mRNAs that correlated statistically significantly with PUS7 and WTAP and that were statistically significantly dysregulated in all three RCC subtypes in comparison to PUS7 high/low and WTAP high/low and which showed correlation coefficients ≥ 0.3 or ≤−0.3 were summarized in Table 3.

This was followed by annotation clustering using the database https://davidbioinformatics.nih.gov/ (accessed on 12 June 2023) [30,31]. The cluster with the highest enrichment score of 5.85 included the following biological functions: mitosis (13 genes), cell cycle (18 genes), and cell division (13 genes). In the context of tumor biology, it is further worth mentioning that the WNT signaling pathway (6 genes) was also identified in another cluster.

## 4. Discussion

The triumph of molecular medicine can be seen in the increasing response rates in the treatment of many pathologies. In particular, in the treatment of various tumor diseases, the steadily increasing overall response rates reflect the progress of basic molecular biology research. Current research is focused on identifying further tumor-biologically relevant pathways that could serve as possible new therapeutic approaches to combine with existing therapies to further increase the overall response rates. Especially in the treatment of RCCs, significant improvements in response rates were seen with the introduction of therapeutic monoclonal antibodies (mAbs) against tumor immune checkpoint axes, which can be combined with each other and are often used in combination with pro-inflammatory cytokines or with receptor tyrosine kinase inhibitors for additive effects. Targeted therapies, such as those against receptor tyrosine kinases or against mTOR, as well as various immunotherapies, are now playing an important role in the treatment of RCCs. These immunotherapies include, in particular, mAbs to inhibit neo-vascularization but also to block inhibitory immune checkpoint (ICP) axes. These mAbs, in combination with each other or with the other forms of therapy previously mentioned, as well as with additive-acting proinflammatory cytokines, show extremely promising overall response rates (ORR) that far outweigh the effects of regular chemotherapy drugs (e.g., nivolumab and cabozantinib) with ORR about 81% [38].

The importance of RNA-modifying factors for translation efficiency and thus for (tumor) cell metabolism (and indirectly for cell migration and invasiveness) and cell proliferation has already been described in the introduction. In fact, the analysis of these RNA-modifying factors in the context of tumor biology is the subject of ongoing research. Such chemical modifications on RNAs are performed by factors that are grouped as either writers, erasers, or readers, where writers induce the modifications, erasers remove these modifications, and readers can interpret the effect of the modified RNA [39].

In particular, pseudouridinylation affects RNA conformation and thus influences its interaction with other RNAs or with RNA-binding proteins. In fact, it is already known that pseudouridinylation of the various RNA species (especially tRNAs, rRNAs, mRNAs, and snRNAs) influences important cellular biological processes, such as alternative splicing and translation [40]. A substitution of uridine with pseudouridine introduces a novel H bond donor on the non-Watson Crick site of the respective nucleotide within the RNA molecule, which affects the secondary structure, predominantly catalyzed by enzymes known as pseudouridine synthases (PUSs), of which 13 PUSs are known in humans, namely: PUS1, PUSL1, PUS3, TRUB1, TRUB2, DKC1, PUS7, PUS7L, RPUSD1, RPUSD2, RPUSD3, RPUSD4, and PUS10 [40]. Recently Li et al. (2023) identified the factor PUS1 and demonstrated that upregulated PUS1 expression resulted in the elevated RCC cancer cell viability, migration, invasion, and colony formation ability in RCC cell lines [24]. In addition, Ding et al. (2024) were able to show that pseudouridylation is a significant biomarker in cancer diagnosis and prognosis in various tumor types, including RCCs. The general overexpression of PUSs is common in cancer cells and predicts poor prognosis [41].

RNA methylations represent another important group of chemical modifications to the respective RNA species. A methyl group can be added at the respective base or, more seldom, even at the ribose residue. More than 72 different RNA methylations are now listed in the online database MODOMICS [42,43]. Fibrillarin (FBL) performs such less frequent 2′-O-methylations by adding a methyl residue to the ribose backbone using S-adenosyl methionine (SAM) as a methyl donor, whereas FBL acts in a complex with other factors including NOP56, NOP58, and 15.5K (SNU13) [44].

The most common RNA methylations localized at the bases include, among others, N6-methyladenosine (m6A), N1-methyladenosine (m1A), and 5-methylcytosine (m5C), besides the 7-methylguanosine m7G [42]. For example, PCIF1 catalyzes the methylation of N6,2′-O-dimethyladenosine (m6Am) in mRNA [45]. RNA methyltransferases (RNMTs) such as METTL3 and METTL14, together as the METTL3-METTL14 complex and with other factors, including WTAP, VIRMA, RBM15, and ZC3H13, form the methyltransferase complex catalyzing the m6A reaction, which occurs in all RNA species [46]. Interestingly, it is the METTL16 methyltransferase that catalyzes m6A methylation in the U6 snRNAs [47], while for 18S rRNA it is METTL5 [7]. In the case of m6A methylation in rRNAs, other RNTMs are known, namely METTL16 (18S rRNA), NSUN2 (28S rRNA), and TRMT10C (5.8S rRNA) [48,49]. Another recently identified human methyltransferase is ZCCHC4, which also catalyzes the methylation at the N6-methyladenosine (m6A) in 28S rRNA specifically on adenosine 1832 (A1832) [50].

The m1A methylation occurs on tRNAs, rRNAs, and mRNAs and involves the following specific methyltransferases: TRMT6, TRMT61A, TRMT61B, TRMT10C, and NML [51]. In the case of m5C RNA methylation, the following factors act as writers: NSUN2, DNMT1, DNMT2, and TRM4 [52]. Furthermore, two other RNMTs, NSUN4 and NSUN5, are known with relevance for m5C methylation, with NSUN4 mainly methylating tRNAs and mRNAs and NSUN5 the 28 S rRNA [53,54]. The complex composed of TRMT6 and TRMT61A methylates tRNAs at cytosine residues, generating m5C modifications [55].

While the writers of the m7G modification include the following factors: RNMT, CMTR1, RAM, WDR4, and METTL1. The m7G modification can occur, e.g., as a 5′-cap on mRNAs but also at internal positions [56]. Given the already large number of RNMTs and other molecules involved and the presence of other less common RNA methylation sites that are therefore not mentioned here, it can be assumed that there are plenty of other relevant RNMTs or molecules involved in these processes with relevance for transcript stability, translation, and thereby indirectly for proliferation and probably tumor biology.

A review article by Alhammadi et al., 2024 [57], was recently published, which discusses the importance of a few individual RNA-modifying factors and brings together actual molecular biological findings of RNA-modifying factors on target genes with high relevance for RCCs. It reveals that, among other things, the mRNA of the prognostic marker PTEN is methylated by METTL14 [58]. In fact, RNA modifications are conceivable for a large number of mRNAs of regulatory target genes—not only tumor suppressors and oncogenes. However, our study directly assesses the entire set of RNA-modifying factors of the writers with direct relations to proliferation markers, prognostic markers, and tumor immune checkpoints in RCC TCGA data sets. For the first time, two RNA-modifying enzymes, namely PUS7 and WTAP, are identified, as being very likely directly involved in the known differences regarding proliferation and malignancy of the three most relevant RCC subtypes—ccRCCs, pRCCs, and chRCCs.

Pseudouridinylation of RNA increases RNA stability and functionality in coding and non-coding RNA species. PUS7 plays an important role in this process because it directly catalyzes this pseudouridinylation, and PUS7 has been recently identified as a promising prognostic marker in tumor diseases themselves [59,60]. The RNA-modifying factor WTAP, which was also identified, plays a very important role as a component of the complex for m6A RNA methylation, since these modifications also play an essential function in all RNA species with regard to RNA stability, splicing, and translation. In fact, the importance of WTAP in tumor biology has already been demonstrated for various tumor entities, including breast cancer and acute myeloid leukemia, where increased WTAP expression was also associated with poorer overall survival [61,62]. The known functions of WTAP in carcinogenesis across various tumor diseases have recently been reviewed by Fan et al. [63].

Interestingly, a direct involvement of WTAP in the m6A methylation of PTEN could actually be demonstrated, which strongly strengthens the quality of our data analysis [64].

In addition, in our analysis, we see a similar, very strong positive correlation between WTAP and NRAS as well as PTEN, in addition to tendencies towards TP53. Such a correlation between TP53 and WTAP in non-small-cell lung cancer has already been described in the literature [65]. Moreover, other recent studies in other tumor entities also demonstrate WTAP itself as a useful prognostic marker [66]. This is very interesting due to the fact that in our analysis these strong positive correlations exist with the prognostic markers that are very important for RCCs. In particular, the non-classical human leukocyte antigen class Ib molecule G (HLA-G), which acts as a ligand for the inhibitory receptors ILT2, ILT4, and KIR2DL4 on all major immune effector cells, can mediate its immunological tolerance both as a membrane-bound molecule and as a secreted molecule. This mechanism can be used by the tumor cells as an immune escape mechanism in a variety of tumor entities, including RCC [67,68,69,70]. As a new finding, in analogy to previous publications, the clinical relevance of HLA-G is once again shown in these RCC data sets, as it correlates statistically significantly with the aggressiveness of the different RCC subtypes. However, only a weak but statistically significant positive correlation with PUS7 and WTAP was found, which could indicate an RNA modification in the form of m6A methylation or pseudouridinylation or a mostly independent behavior of RNA modifications. However, there are no publications on this in the literature yet. It is also worth noting that HLA-G is the only representative of the HLA molecules that is strongly characterized by alternative splicing. There are 7 different HLA-G splice variants, so an accurate HLA-G mRNA modification is essential for this alternative splicing per se [71].

Galectin-9 (LGALS9) also inhibits the anti-tumor effect of T and NK cells, where it binds to TIM-3 as an inhibitory ligand. In addition, the relevance of this immunosuppressive molecule in ccRCCs has already been demonstrated [63], and, in analogy to HLA-G, there are already mAbs against this tumor immune checkpoint for anti-tumor therapies [71,72,73].

Surprisingly, only one of the known proliferation markers relevant for RCC, CXCR4, showed expression data that match the different proliferations of the RCC subtypes. This suggests that even better proliferation markers could be found for RCCs or only a combined usage of those markers reveals the proliferative abilities of RCC. Furthermore, no RNA modifications such as m6A methylation or pseudouridinylation are known for CXCR4. At this point, it should be explicitly pointed out that the known proliferation markers at the protein level may well be suitable for use in everyday clinical practice, even if these markers could not be identified at the transcript level in this study. This is an important limitation of this study.

Table 3 summarizes the putative genes that correlate statistically significantly with PUS7 and WTAP. In fact, there are individual genes among them with very well-known connections to solid and hematopoietic tumor diseases per se—see the following selected examples. The gene name *MKI67* encodes what is probably the best-known proliferation marker and is very frequently used in pathological studies in various tumor diseases. Altered expression of *EPHA3* also correlates with tumor diseases [74], which is also true for the potential oncogene *CREB5* [75]. *CDH13* expression is physiologically atypical, and reexpression is observed in various tumor diseases, including even in ccRCC [76]. Additionally, *MELK* has been characterized as an oncogenic kinase essential for metastasis and mitotic progression in lung carcinoma [77].

In particular, the altered molecular properties, which form the basis for enabling altered proliferation kinetics of tumor cells, should be of greater interest, as this could conceal so far putative unused therapeutic options that could target the causes (an establishing tumor cell) rather than the consequences (an already established tumor cell). An increase in the speed of the cell cycle in tumor cells inevitably leads to an increase in errors in DNA replication and resistance to apoptosis. In combination with the ability to evade immune effector cells, even allowing the persistence of such cancer cells, which, through ongoing neoangiogenesis, deprive even the last healthy cells of nutrients and, if left untreated, not only result in the inevitable tissue death but also culminate in the death of the entire patient.

The identification of chemical modifications of individual coding mRNAs and their aberrations in various pathologies, including tumor diseases, is currently still in its beginnings. Therefore, such initial analyses are indispensable for identifying direct target mRNAs for further separate analyses. However, these initial in silico studies based on transcriptome data cannot replace laboratory research. Rather, the aim is to sensitize the attention of current laboratory research to RNA-modifying enzymes. Overexpression or knockdown/out studies in human cells should follow to validate the molecular relationships formulated here.

In selected tumor entities, two important new central target structures, PUS7 and WTAP, were identified that directly control essential points for proliferation, which was impressively supported by annotation clustering. However, the results of the Kaplan–Meier curves and the heat maps allow the conclusion that there is subtype-specific relevance even within RCCs and that the two factors are particularly important for ccRCCs. In this context, RNA-modifying enzymes should be highlighted as a completely new approach to controlling tumor cell proliferation as a therapeutic option.

## 5. Conclusions

Gene therapies are rapidly becoming part of the clinical treatment spectrum for a variety of different genetic diseases. For example, antisense oligonucleotides can be used to induce exon skipping in patients with Duchenne muscular dystrophy [78]. Casgevy^®^ (Exagamglogene autotem-cel), the first CRISPR/Cas9-based gene therapy, has now found its way into clinical therapies [79]. This technique is based on the knockout of defective genes. In contrast, gene replacement preparations that can introduce an intact gene into affected patients have been on the market for some time. These now include 9 different available preparations: Glybera^®^, Luxturna^®^, Zolgensma^®^, Upstaza^®^, Roctavian^®^, Hemgenix^®^, Libmeldy^®^, Zynteglo^®^, and Skysona^®^ [80]. There are now also 6 different siRNA-based therapies available: Patisiran^®^, Givosiran^®^, Lumasiran^®^, Inclisiran^®^, Nedosiran^®^, and Vutisiran^®^ [81]. The range of diseases that can be treated with such gene therapies is very large and heterogeneous. Sooner or later, the first anti-tumoral gene therapies will begin their triumphant advance.

The introduction already pointed out the large number of dysregulated genes involved in the various solid and hematopoietic tumor diseases, so that combination therapies will inevitably be the key to success. This, in turn, requires a comprehensive analysis of the molecular relationships. This in silico analysis based on statistical evaluations of clinical TCGA data sets from healthy kidney tissue and the three most common RCC subtypes led to the identification of two RNA-modifying proteins—PUS7 and WTAP. Nevertheless, it should be explicitly pointed out that such initial in silico analyses cannot replace any validations of the obtained results using standard molecular biology methods, for example, to assess whether the observed correlations are direct or indirect effects. This represents an important limitation of the obtained findings and performed statements but opens up new routes for future research.

## Figures and Tables

**Table 1:** Summary of the analyzed genes and their functions.

Proliferation Marker	Prognosis Marker	Tumor Immune Checkpoints	RNA Pseudouridinylation	RNA Ortho-Methylation	RNA N-Methylation
MKI67	TP53	CD47	PUS1	FBL	PCIF1
PCNA	BCL2	CD24	PUSL1	NOP56	METTL3
MCM2	BIRC5	STC1	PUS3	NOP58	METTL14
MCM4	PTEN	CD274	TRUB1	SNU13	WTAP
CENPF	NRAS	HLA-G	TRUB2		VIRMA
CXCR4	TSC1	HLA-E	DKC1		RBM15
	TSC2	LGALS3	PUS7		ZC3H13
	CDKN2A	FGL1	PUS7L		METTL16
		LGALS9	RPUSD1		METTL5
		HMGB1	RPUSD2		NSUN2
		PVR	RPUSD3		TRMT10C
			RPUSD4		ZCCHC4
			PUS10		TRMT6
					TRMT61A
					TRMT61B
					RRP8
					DNMT1
					TRDMT1
					NSUN4
					NSUN5
					RNMT
					CMTR1
					WDR4
					METTL1

**Table 2:** Correlation of PUS7 and WTAP with other statistically significant dysregulated tumor-relevant putative target mRNAs in the RCC subtypes.

	ccRCC		pRCC		chRCC		ΣRCC		Non-Tumorous Kidney	
	PUS7	WTAP	PUS7	WTAP	PUS7	WTAP	PUS7	WTAP	PUS7	WTAP
CXCR4	R = 0.4	R = 0.4	R = 0.1	R = 0.4	R = 0.4	R = 0.5	R = 0.5	R = 0.5	R = 0.31	R = 0.42
	[0.32; 0.47]	[0.29; 0.44]	[0.02; 0.23]	[0.28; 0.47]	[0.25; 0.60]	[0.26; 0.61]	[0.48; 0.57]	[0.47; 0.57]	[0.12; 0.49]	[0.23; 0.58]
	*p* < 1.0 × 10^−10^	*p* < 1.0 × 10^−10^	*p* = 0.0273	*p* < 1.0 × 10^−10^	*p* = 1.4 × 10^−5^	*p* = 8.6 × 10^−6^	*p* < 1.0 × 10^−10^	*p* < 1.0 × 10^−10^	*p* = 0.0013	*p* = 1.2 × 10^−5^
TP53	R = 0.5	R = 0.5	R = 0.6	R = 0.5	R = 0.7	R = 0.8	R = 0.5	R = 0.5	R = 0.61	R = 0.50
	[0.37; 0.52]	[0.47; 0.60]	[0.51; 0.66]	[0.41; 0.58]	[0.53; 0.80]	[0.67; 0.87]	[0.58; 0.61]	[0.59; 0.61]	[0.45; 0.74]	[0.31; 0.66]
	*p* < 1.0 × 10^−10^	*p* < 1.0 × 10^−10^	*p* < 1.0 × 10^−10^	*p* < 1.0 × 10^−10^	*p* < 1.0 × 10^−10^	*p* < 1.0 × 10^−10^	*p* < 1.0 × 10^−10^	*p* < 1.0 × 10^−10^	*p* < 1.0 × 10^−10^	*p* = 1.6 × 10^−6^
PTEN	R = 0.5	R = 0.6	R = 0.6	R = 0.6	R = 0.8	R = 0.9	R = 0.7	R = 0.7	R = 0.74	R = 0.80
	[0.45; 0.58]	[0.55; 0.66]	[0.50; 0.67]	[0.56; 0.71]	[0.71; 0.87]	[0.83; 0.92]	[0.63; 0.71]	[0.67; 0.74]	[0.64; 0.82]	[0.70; 0.87]
	*p* < 1.0 × 10^−10^	*p* < 1.0 × 10^−10^	*p* < 1.0 × 10^−10^	*p* < 1.0 × 10^−10^	*p* < 1.0 × 10^−10^	*p* < 1.0 × 10^−10^	*p* < 1.0 × 10^−10^	*p* < 1.0 × 10^−10^	*p* < 1.0 × 10^−10^	*p* < 1.0 × 10^−10^
NRAS	R = 0.7	R = 0.7	R = 0.6	R = 0.6	R = 0.8	R = 0.9	R = 0.73	R = 0.7	R = 0.62	R = 0.66
	[0.61; 0.71]	[0.62; 0.71]	[0.50; 0.66]	[0.49; 0.65]	[0.65; 0.83]	[0.87; 0.95]	[0.70; 0.76]	[0.70; 0.76]	[0.46; 0.73]	[0.51; 0.78]
	*p* < 1.0 × 10^−10^	*p* < 1.0 × 10^−10^	*p* < 1.0 × 10^−10^	*p* < 1.0 × 10^−10^	*p* < 1.0 × 10^−10^	*p* < 1.0 × 10^−10^	*p* < 1.0 × 10^−10^	*p* < 1.0 × 10^−10^	*p* < 1.0 × 10^−10^	*p* < 1.0 × 10^−10^
HLA-G	R = 0.2	R = 0.2	R = 0	R = 0.2	R = 0.3	R = 0.3	R = 0.3	R = 0.4	R = 0.38	R = 0.41
	[0.09; 0.25]	[0.16; 0.32]	[−0.14; 0.08]	[0.08; 0.30]	[0.05; 0.44]	[0.14; 0.52]	[0.22; 0.34]	[0.30; 0.41]	[0.19; 0.55]	[0.22; 0.58]
	*p* = 3.9 × 10^−5^	*p* = 6.2 × 10^−9^	*p* = 0.6350	*p* = 0.0004	*p* = 0.0168	*p* = 0.0010	*p* < 1.0 × 10^−10^	*p* < 1.0 × 10^−10^	*p* = 8.2 × 10^−5^	*p* = 1.6 × 10^−5^
LGALS9	R = 0.2	R = 0.3	R = 0.1	R = 0.2	R = 0.4	R = 0.4	R = 0.4	R = 0.4	R = 0.49	R = 0.43
	[0.14; 0.31]	[0.17; 0.33]	[−0.04; 0.18]	[0.13; 0.34]	[0.20; 0.56]	[0.26; 0.59]	[0.29; 0.41]	[0.32; 0.44]	[0.32; 0.62]	[0.26; 0.58]
	*p* = 5.5 × 10^−8^	*p* = 3.1 × 10^−10^	*p* = 0.2035	*p* = 1.1 × 10^−5^	*p* = 0.0001	*p* = 1.9 × 10^−5^	*p* < 1.0 × 10^−10^	*p* < 1.0 × 10^−10^	*p* = 3.0 × 10^−7^	*p* = 6.5 × 10^−6^

Numbers in brackets correspond to the lower and upper values of the 95% confidence interval, respectively.

**Table 3:** Results of transcriptome analysis of dysregulated genes with statistically significant correlations to PUS7 and WTAP.

PUS7				WTAP			
Gene	Expression	Correlation Coefficient	*p*-Value	Gene	Expression	Correlation Coefficient	*p*-Value
*ACLY*	190.0238	0.7465	4.68 × 10^−178^	*KLF10*	687.2348	0.5013	1.73 × 10^−64^
*NUF2*	179.1750	−0.3738	2.20 × 10^−34^	*FRZB*	119.7849	0.6163	2.97 × 10^−105^
*SST*	140.5689	0.6030	1.20 × 10^−99^	*CPA3*	104.5162	0.6406	3.96 × 10^−116^
*NPTX2*	111.1566	0.5450	3.81 × 10^−78^	*CLEC4E*	103.6079	0.5289	7.08 × 10^−73^
*MELK*	103.6079	0.4901	2.56 × 10^−61^	*RAPGEF5*	75.9460	0.5316	9.49 × 10^−74^
*HAVCR1*	78.9817	0.4859	3.91 × 10^−60^	*POSTN*	75.4483	0.3515	2.45 × 10^−30^
*REL*	78.8732	0.5333	2.88 × 10^−74^	*EPHA3*	73.3262	0.4336	6.45 × 10^−47^
*TNFAIP6*	77.3074	0.5625	3.41 × 10^−84^	*ARHGAP29*	53.2551	0.6308	1.29 × 10^−111^
*IL1RAP*	75.8770	0.5011	1.90 × 10^−64^	*IFI44L*	47.7831	0.4633	3.90 × 10^−54^
*VCAN*	73.3262	0.4964	4.26 × 10^−63^	*ADGRG6*	44.4220	0.7105	7.15 × 10^−154^
*EDN1*	66.8430	0.6020	3.08 × 10^−99^	*SFRP2*	29.5562	0.6880	1.39 × 10^−140^
*SCGN*	62.5494	0.4680	2.39 × 10^−55^	*ITGA4*	24.6503	0.4976	1.99 × 10^−63^
*CREB5*	60.7807	0.7228	1.04 × 10^−161^	*SCARA3*	22.4505	0.4419	7.25 × 10^−49^
*PBK*	55.8713	0.5313	1.21 × 10^−73^	*CDH13*	17.8840	0.6109	6.22 × 10^−103^
*ARHGAP11A*	50.1871	0.4210	4.64 × 10^−44^	*IRAK3*	14.5509	0.5764	2.98 × 10^−89^
*FRZB*	40.2235	0.6295	4.95 × 10^−111^	*RGS5*	11.9904	0.6551	3.70 × 10^−123^
*LOXL2*	39.4410	0.6048	2.12 × 10^−100^	*ECEL1*	10.0061	0.6368	2.32 × 10^−114^
*QRFPR*	28.1993	0.5818	2.69 × 10^−91^	*VNN2*	9.5169	0.4689	1.36 × 10^−55^
*COL5A1*	25.9432	0.5086	1.20 × 10^−66^	*GJA1*	9.2066	0.5597	3.32 × 10^−83^
*SLC5A1*	24.9524	0.3168	1.20 × 10^−24^	*ITGB6*	8.5897	0.5524	1.23 × 10^−80^
*ADGRG6*	24.6503	0.5560	6.72 × 10^−82^	*INHA*	7.9855	0.6423	5.94 × 10^−117^
*SLC7A2*	20.7983	0.4573	1.23 × 10^−52^	*NID2*	6.4530	0.5376	1.15 × 10^−75^
*RECQL*	20.2517	0.6875	2.65 × 10^−140^	*APOLD1*	5.9691	0.5043	2.35 × 10^−65^
*RRM2*	19.5924	−0.4178	2.35 × 10^−43^	*TGFB2*	3.8323	0.6762	4.63 × 10^−134^
*RAPGEF5*	17.8840	0.6231	3.42 × 10^−108^	*OLFM4*	3.6288	0.5691	1.44 × 10^−86^
*ZC3HAV1L*	15.7110	0.4402	1.83 × 10^−48^	*AC003092.1*	2.0515	0.4798	1.76 × 10^−58^
*NID2*	14.5509	0.5850	1.68 × 10^−92^	*ELK3*	1.8317	0.4500	8.06 × 10^−51^
*FAM111B*	14.5212	0.5169	4.01 × 10^−69^				
*MKI67*	14.0369	−0.4819	4.80 × 10^−59^				
*SSPN*	13.6805	0.7057	6.72 × 10^−151^				
*GAS2L3*	13.2700	0.6528	5.13 × 10^−122^				
*P4HA3*	11.7873	0.7959	7.82 × 10^−219^				
*LRRK2*	11.1293	0.6397	9.65 × 10^−116^				
*TPX2*	11.0676	0.5789	3.60 × 10^−90^				
*TOP2A*	10.5442	0.6520	1.33 × 10^−121^				
*PREX2*	10.0566	0.5676	5.03 × 10^−86^				
*KCNK3*	8.0663	0.6998	2.28 × 10^−147^				
*EPHA3*	6.4530	0.5480	3.85 × 10^−79^				
*IL18R1*	5.7964	0.6681	9.00 × 10^−130^				
*FRMD6*	5.6745	0.6742	5.56 × 10^−133^				
*ENPP3*	5.4524	0.6710	2.93 × 10^−131^				
*OSMR*	5.0688	0.6223	7.87 × 10^−108^				
*ANLN*	5.0446	0.6949	1.54 × 10^−144^				
*FOXM1*	4.9233	0.5749	1.13 × 10^−88^				
*ARL4C*	4.7428	0.5523	1.31 × 10^−80^				
*CDON*	4.6661	0.6809	1.28 × 10^−136^				
*SACS*	4.4960	0.6107	7.38 × 10^−103^				
*CEP55*	4.3923	0.6164	2.75 × 10^−105^				
*TNNT1*	4.3796	0.3098	1.34 × 10^−23^				
*CDCA7*	4.2515	0.5939	5.30 × 10^−96^				
*PGM2L1*	4.0652	−0.3533	1.20 × 10^−30^				
*SEMA3D*	4.0290	0.5363	2.90 × 10^−75^				
*AGMO*	4.0251	0.6211	2.55 × 10^−107^				
*KIF20B*	3.8307	0.7649	4.79 × 10^−192^				
*NTM*	3.6732	0.5229	5.31 × 10^−71^				
*BUB1*	3.5337	0.5834	6.58 × 10^−92^				
*BUB1B*	3.5335	0.3494	5.62 × 10^−30^				
*MALL*	3.4257	0.6727	3.52 × 10^−132^				
*CENPF*	3.2824	0.6335	7.55 × 10^−113^				
*USP37*	2.9702	0.6771	1.44 × 10^−134^				
*PRR11*	2.9327	0.6409	2.59 × 10^−116^				
*KIF4A*	2.9095	0.6239	1.45 × 10^−108^				
*CCNA2*	2.8841	0.5970	3.13 × 10^−97^				
*CDCA2*	2.6303	0.5656	2.82 × 10^−85^				
*HMMR*	2.5460	0.5881	1.02 × 10^−93^				
*BRCA1*	2.4850	0.7127	3.06 × 10^−155^				
*DLGAP5*	2.4775	0.6151	9.80 × 10^−105^				
*NEK2*	2.2389	0.6021	2.72 × 10^−99^				
*XIST*	2.0515	0.4606	1.91 × 10^−53^				
*FZD1*	2.0050	0.6155	6.59 × 10^−105^				
*NCAPG*	1.9583	0.6394	1.41 × 10^−115^				
*EDIL3*	1.8830	0.5784	5.38 × 10^−90^				
*DTL*	1.8648	0.6236	2.12 × 10^−108^				
*DKK1*	1.8317	0.4001	1.38 × 10^−39^				
*CENPK*	1.7248	0.6565	7.56 × 10^−124^				

## Data Availability

The original contributions presented in this study are included in the article/Supplementary Material. Further inquiries can be directed to the corresponding author.

## Supplementary Materials

The following supporting information can be downloaded at https://www.mdpi.com/article/10.3390/cimb47040266/s1.

## Abbreviations

The following abbreviations are used in this manuscript:

AS	Alternative splicing
DNA	Deoxyribonucleic acid
ccRCC	Clear cell RCC
CDS	Coding sequence
chRCC	Chromophobe RCC
HLA	Human leukocyte antigen
FDR	False discovery rate
ICP	Immune checkpoint
lncRNA	Long non-coding RNA
mAb	Monoclonal antibody
mRNA	Messenger RNA
miRNA	Micro RNA
PAP	Poly(A) polymerase
ORR	Overall response rate
pRCC	Papillary RCC
RCC	Renal cell carcinoma
RNA	Ribonucleic acid
RNMTs	RNA methyltransferases
rRNA	Ribosomal RNA
snRNA	Small nuclear RNA
TE	Translational efficiency
TPM	Transcripts per million
tRNA	Transfer RNA
UTR	Untranslated region
